# The evolution of gastrulation morphologies

**DOI:** 10.1242/dev.200885

**Published:** 2023-04-17

**Authors:** Guillermo Serrano Nájera, Cornelis J. Weijer

**Affiliations:** ^1^ Department of Genetics, University of Cambridge, Cambridge, CB2 3EH, UK; ^2^ School of Life Sciences Research Complex, University of Dundee, Dow Street, Dundee, DD1 5EH, UK

**Keywords:** Gastrulation, Evolution, EMT, Cell behaviours, Yolk, Morphogenesis

## Abstract

During gastrulation, early embryos specify and reorganise the topology of their germ layers. Surprisingly, this fundamental and early process does not appear to be rigidly constrained by evolutionary pressures; instead, the morphology of gastrulation is highly variable throughout the animal kingdom. Recent experimental results demonstrate that it is possible to generate different alternative gastrulation modes in single organisms, such as in early cnidarian, arthropod and vertebrate embryos. Here, we review the mechanisms that underlie the plasticity of vertebrate gastrulation both when experimentally manipulated and during evolution. Using the insights obtained from these experiments we discuss the effects of the increase in yolk volume on the morphology of gastrulation and provide new insights into two crucial innovations during amniote gastrulation: the transition from a ring-shaped mesoderm domain in anamniotes to a crescent-shaped domain in amniotes, and the evolution of the reptilian blastoporal plate/canal into the avian primitive streak.

## Teaser

**Summary:** This Hypothesis reviews the mechanisms that underlie the plasticity of vertebrate gastrulation during evolution and under experimental manipulation, and discusses the effects of the increase in yolk volume on the morphology of gastrulation.

## Introduction

The early steps of development after fertilisation vary significantly throughout the animal kingdom. However, early development typically results in the generation of an epithelial cell layer composed of pluripotent progenitors, which execute gastrulation. Gastrulation is a morphogenetic programme, during which the embryo transforms from a single one-dimensional epithelial cell layer (the blastula) into an organised, three-dimensional gastrula consisting of the germ layers (ectoderm, mesoderm and endoderm in triploblastic species). Gastrulation arises from coordinating a set of characteristic epithelial cell behaviours: division, shape change, division, as well as cell rearrangement and/or abandoning the layer via ingression. Through the spatiotemporal coordination of these cell behaviours, epithelial layers change shape, bend, flow, grow and shrink during morphogenesis (Collinet and Lecuit, 2021), giving rise to the tissue movements that segregate the germ layers previously specified by specific gene regulatory networks (GRNs).

Genetic evidence suggests that most of the molecular toolkit necessary to create epithelial layers is already present in sponges (Ereskovsky et al., 2013) and probably precedes the evolution of metazoans (Renard et al., 2021). However, although the formation of some sponge larvae requires morphogenetic movements reminiscent of gastrulation, they are not associated with the segregation of proper embryonic layers with restricted cell fates (Ereskovsky and Dondua, 2006; Nakanishi et al., 2014). Cnidarians, on the other hand, present bona fide epithelial cells and gastrulation movements (Renard et al., 2021). Therefore, it seems that all animals share the capacity for complex morphogenetic movements, and gastrulation appeared when morphogenetic movements in the early embryo became associated with the patterning and segregation of the embryonic layers in eumetazoans.

## Mesendoderm internalisation is a major determinant of gastrulation mode

**Fig. 1.**
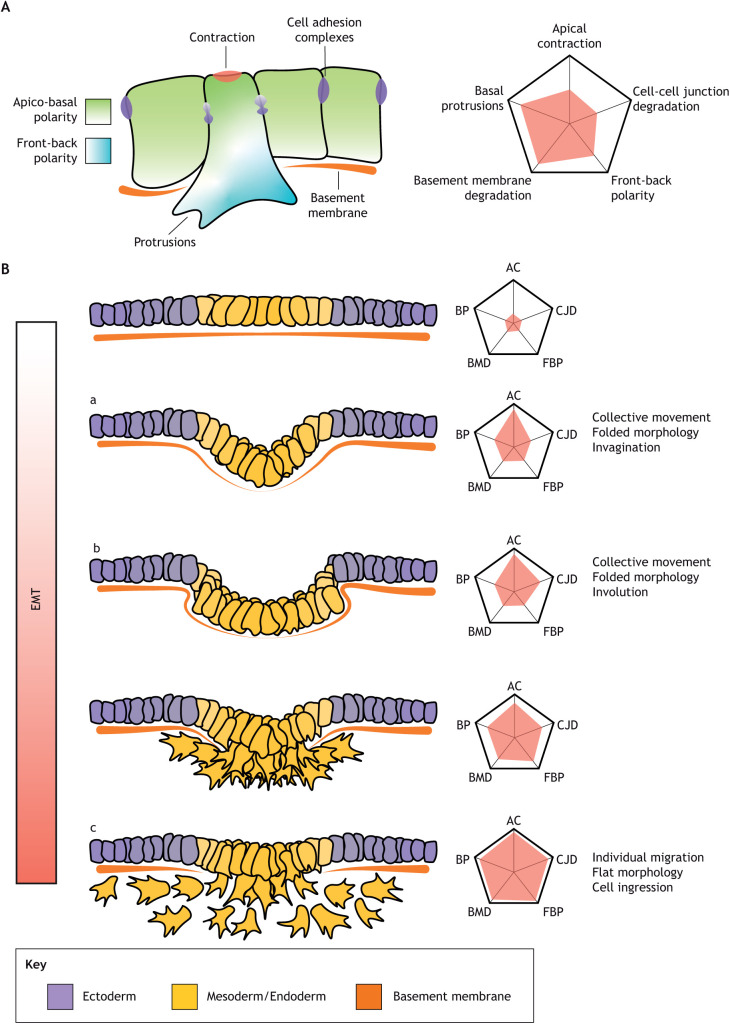
**Different degrees of EMT correlate with different tissue morphologies.** (A) EMT is a gradual, multifactorial process in which different cellular requirements can be independently regulated. (B) The degree of EMT in a cell population determines tissue morphology. Slow or incomplete EMT produces tissue folding in the form of invagination (Ba) where cells apically contract, resulting in localised tissue folding in a confined area in which cells do not slide past each other and can maintain junctional contacts. During involution (Bb) cells also remain part of an epithelial layer, but cells slide against each other through successive rows of cells. Fast complete EMT results in flat epithelia and independent cell migration (ingression; Bc), these cells slide past each other until they lose contact.

Gastrulation is a morphogenetic programme that needs to accomplish three objectives: the generation of the main body axis, the patterning of the germ layers and the internalisation of the mesodermal and endodermal precursors. The mode of internalisation largely determines morphogenesis during gastrulation. Most invertebrates and anamniotes internalise the mesoderm as a continuous epithelial layer by bending the blastoderm inwards (invagination), as in sea urchins (McClay et al., 2020) and *Drosophila melanogaster* (Gheisari et al., 2020), or by rolling through a slit-shaped opening (involution) as in *Xenopus* (Winklbauer, 2020). In other organisms (including all amniotes such as reptiles, birds and mammals), mesoderm precursors acquire a mesenchymal phenotype and ingress into the embryo as individual cells, such as in mouse (Williams et al., 2012) and chick (Stern, 2004). While involution or invagination (where the mesoderm precursors move as a coherent epithelial layer) require the folding of the epiblast, ingression allows the epiblast to remain flat. The internalisation of the mesodermal and endodermal epithelia by invagination, involution or ingression requires a great variety of coordinated structural changes described as an epithelial-to-mesenchymal transition (EMT) (Fig. 1A). Features of EMT include replacing the characteristic apicobasal polarity of epithelial cells with front-back polarity, dismantling tight and adherens junctions, and the acquisition of an irregular and motile morphology (Gonzalez and Medici, 2014). Rather than EMT being just a binary switch between an epithelial or mesenchymal state, it is now understood that different combinations of adhesion, polarity and cytoskeletal components can result in a spectrum of states from fully epithelial to fully mesenchymal (Campbell and Casanova, 2016; Plygawko et al., 2020).

Fully epithelial cells display only limited movement capabilities, whereas fully mesenchymal cells [e.g. chicken mesoderm (Yang et al., 2008)] are highly motile and can migrate as individuals over large distances. The states in between manifest a gradient of migration modes from more coherent as the cells present more epithelial components [e.g. *Xenopus* internalisation (Winklbauer, 2020)] to more disorganised when cells possess a more mesenchymal phenotype [e.g. as occurs in zebrafish (Pinheiro and Heisenberg, 2020)]. The patterning of the mesoderm and endoderm, and the extent to which mesoderm precursors undergo EMT, are major determinants of gastrulation morphology (Fig. 1B). Remarkably, the initial cell shape changes required for invagination of the tissue and individual cell ingression are very similar: apical contraction, apicobasal shortening and basal expansion. Therefore, the difference between cell ingression or invagination could be caused by quantitative differences in the degree of the expression of the mesenchymal programme (Nakaya and Sheng, 2008). If cells maintain their cell-cell adhesion and apicobasal polarity, the coordinated contraction of the cells results in tissue bending producing invagination (Fig. 1Ba) or involution (Fig. 1Bb). However, if they continue transitioning towards a mesenchymal phenotype, with loosening of cell contacts and increased motility, it will result in individual cell ingressions (Fig. 1Bc). Involution is likely caused by a process known as telescoping, where neighbouring cells may or may not apically contract but undergo a partial EMT and partially glide over each other in a wave-like process but remain part of a continuous epithelial layer (Winklbauer, 2020). Whereas organisms that internalise the tissue primarily by cell ingression retain a flat blastoderm, those which use involution undergo dramatic morphogenetic changes that fold the blastoderm inwards. Moreover, the mode of internalisation and the degree of EMT have major consequences for the generation of stress patterns and tissue material properties driving gastrulation (Box 1).

**Box 1. Internalisation mechanisms and mechanics**

The mode of internalisation and epithelial-to-mesenchymal transition (EMT) likely shape the tissue scale forces that drive the movement of the cells in the epiblast and the internalising mesoderm and endoderm. For example, invagination of the mesoderm in *Drosophila* appears to be driven by apical contraction of the mesodermal cells driving tube formation through initial buckling. At the same time, apical-basal shortening of the surrounding ectoderm exerts pressure that helps to complete invagination (Fierling et al., 2022; Guo et al., 2022). Once the cells undergo EMT, they migrate along the inside of the epithelial wall (Clark et al., 2011), exerting a traction force on the overlying epithelium that is insufficient to affect its shape or cellular behaviours. In *Xenopus*, the involuting leading edge mesoderm cells expressing *goosecoid* migrate collectively towards the animal pole on the overlaying blastocoel roof guided by PDGF signalling (Nagel et al., 2004; Winklbauer, 2020). These leading-edge mesoderm cells pull in concert with actively migrating monopolar endoderm cells on the following *brachyury (tbxt*)-expressing mesoderm that undergoes active mediolateral intercalation. This movement pulls on the involuting apical mesoderm and helps the mesoderm invagination process, which is pushed by the radial intercalation-driven epiboly (Keller, 1980; Winklbauer, 2020). This internalisation mode is a push-pull mechanism involving cells on the surface and internalised mesoderm and endoderm cells. An epiblast push-pull mechanism is provided by streak elongation in the chick embryo. During the extension of the streak, the mesendoderm cells ingress and move away as individual cells. However, the apical contraction and ingression of these cells in the central midline pulls neighbouring epiblast cells towards the streak (Rozbicki et al., 2015). This pulling force generates myosin cables aligned perpendicular to the extending streak, which in turn direct intercalations perpendicular to the streak in tissue adjacent to the streak, driving its extension (Chuai et al., 2023; Serra et al., 2021 preprint).

## Experimental synthesis of alternative gastrulation modes

The large morphological variations, and their associated internalisation methods due to wide variations in gastrulation mechanisms among related organisms, suggest that transitioning from one gastrulation mode to another does not necessarily present a strong evolutionary constraint. Indeed, recent experiments have demonstrated that it is possible to generate different gastrulation modes in single organisms by performing perturbations of a small number of components that change the mode of internalisation. Changing the gene expression, the signalling to specific cell behaviours or the geometry of the embryo makes it possible to transition from gastrulation modes based on tissue invagination to modes using cell ingression across the animal kingdom.

**Fig. 2.**
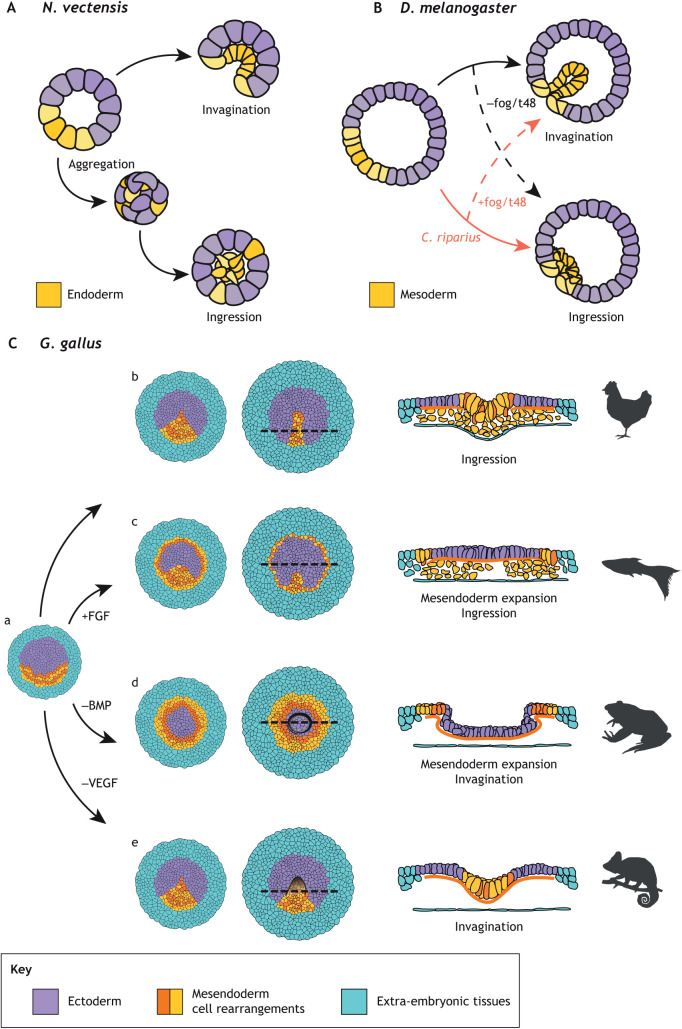
**Experimental manipulation of the gastrulation mode in different organisms.** (A) The anemone *N. vectensis* normally gastrulates by invaginating the endoderm. However, when cells are dissociated and reaggregated into a solid ball, it gastrulates by multipolar cell ingression (Kirillova et al., 2018). (B) The fly *D. melanogaster* has a maternal load of *fog*/*t48* mRNAs and normally gastrulates through invagination, whereas *C. riparius* is not loaded with *fog*/*t48* mRNAs and gastrulates through cell ingression. Switching the typical *fog* expression of both species also switches their gastrulation mode (Urbansky et al., 2016). (C) The activation or inhibition of signalling pathways in the chick embryo (Ca) affects the extension of the mesendoderm and the capacity of these cells to ingress (Cb) inducing gastrulation modes that resemble those of other vertebrates such as teleost fish (Cc), anurans (Cd) and reptiles (Ce) (Chuai et al., 2023).

In the sea anemone, *Nematostella vectensis*, endoderm cells undergo a partial EMT programme and are internalised by invagination (Kraus and Technau, 2005; Magie et al., 2007; reviewed by Technau, 2020). However, direct disruption of the PAR polarity complex leads to the disassembly of the adherens junctions in the endodermal plate, where some cells acquire a mesenchymal phenotype and appear to be internalised by cell ingression rather than by invagination (Salinas-Saavedra et al., 2018). A more comprehensive phenotype occurs when the cells in the early embryo are dissociated and reaggregated, but *N. vectensis* can still develop to adult stages (Kirillova et al., 2018). In the aggregates, the embryonic geometry has been altered from a hollow sphere with polarised endoderm to a compact ball in which endoderm and ectoderm are mixed. Surprisingly, instead of using the coherent invagination of the endoderm at one pole, the embryos use ingression from distinct sites (multipolar ingression) and cavitation (Fig. 2A), a gastrulation mode typical of other cnidarians (Kirillova et al., 2018), demonstrating that *N. vectensis* can access alternative developmental trajectories when the patterning and the topology of the embryo are altered.

Another recent example uses different fly species that exhibit different gastrulation modes. Whereas some flies internalise the mesendoderm using a prominent invagination (*Drosophila*, *Megaselia*), gastrulation in basal species of flies (*Anopheles*, *Clogmia*, *Chironomus*) is flat or forms just a shallow groove (Lemke et al., 2020). Interestingly, it is possible to transition from invagination to flat gastrulation modes and vice versa in different species by modifying the maternal loading of either *Fog* or *t48* mRNA (Urbansky et al., 2016) (Fig. 2B), two genes that modulate the level of EMT in mesoderm cells (Weng and Wieschaus, 2016). This demonstrates that relatively small changes in the loading of maternal mRNAs can have a substantial impact on the gastrulation morphology of flies, probably through the regulation of the degree of EMT in mesoderm cells.

Finally, by interfering with crucial developmental signalling pathways it is possible to change the patterning and the internalisation mode of the mesoderm during chick gastrulation (Chuai et al., 2023) (Fig. 2C and see below). Doing so results in major morphogenetic changes that reproduce key aspects of gastrulation in other vertebrates. Altogether, these experiments suggest (1) that the mode of internalisation is a major determinant in gastrulation morphology, (2) that transitioning between different internalisation modes can be achieved with relatively small changes, and (3) that these observations generally apply across the animal kingdom.

Here, we use the experimental generation of alternative gastrulation modes in chick embryos to discuss the three axes of variation in gastrulation modes in early vertebrate embryos: mesoderm internalisation, the spatial pattern of mesoderm induction and the embryo's geometry. We review previously unrecognised evolutionary constraints revealed by these experiments and propose that increased yolk volume during evolutionary time has driven two crucial innovations in the evolution of amniote gastrulation: the mesoderm ring-crescent transition and the blastopore-primitive streak transition. Finally, we comment on the evolvability of gastrulation.

## Synthesis of vertebrate gastrulation modes

**Fig. 3.**
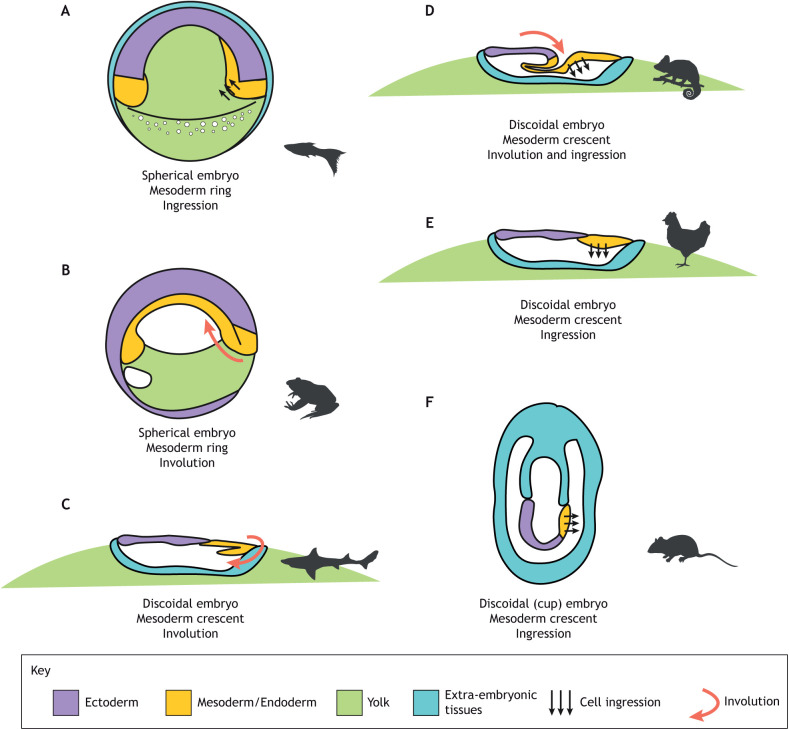
**Diversity of vertebrate gastrulation.** (A-F) Schematic representations of the main axis of variation in vertebrate gastrulation (geometry of the embryo, shape of the mesoderm/endoderm territory and the main mode of internalisation) in teleost fish (A), anurans (B), cartilaginous fishes (C), reptiles (D), birds (E) and rodents (F). Most mammals, such as human (Molè et al., 2020), cow (van Leeuwen et al., 2015), rabbit (Viebahn et al., 2002) or pigs (Wolf et al., 2011), possess a flat epiblast and primitive streak similar to the early embryos of birds (E); however, mouse presents a more derived gastrulation where the flat epiblast is folded into a cup and the primitive streak is specified in place (Williams et al., 2012) (F).

Gastrulation morphology is highly variable among vertebrates (Fig. 3). One axis of variation is the general geometry: whereas some embryos are spherical as in teleost fish (Fig. 3A) and amphibians (Fig. 3B), other embryos possess a flat blastoderm as in cartilaginous fish (Fig. 3C) and amniote embryos (Fig. 3D-F). A second source of variability is the location of the presumptive mesoderm. In spherical embryos a ring forms between the animal and vegetal poles, whereas in flat embryos the mesoderm is restricted to a crescent at the posterior end of the blastoderm. Finally, as discussed above, embryos differ in their internalisation mode; however, there is no apparent link between the geometry and the internalisation mode, because both ingression and involution can be found in spherical and flat embryos. Recent experimental results using chick embryos (Chuai et al., 2023), a flat embryo that internalises mesoderm through cell ingression, show that it is possible to modify the shape of the mesoderm (from a crescent to a ring) and its internalisation mode, generating gastrulation modes that resemble those of other vertebrates. Here, we propose that these results in chick gastrulation explain why the flat embryos always seem to be associated with a posterior crescent of mesoderm.

### Crescent-shaped mesoderm with cell ingressions

Before the onset of chick gastrulation, the mesoderm is specified as a crescent at the posterior border of the epiblast by signals from the extra-embryonic tissues (Fig. 2Ca). During normal gastrulation, cells in the mesoderm territory undergo oriented cell intercalations, producing convergent extension of the tissue (Rozbicki et al., 2015; Voiculescu et al., 2007). The process of convergent extension is dependent on the generation of supra-cellular myosin cables spanning between 2 and 8 cells that extend along the long axis of the mesoderm crescent (Rozbicki et al., 2015). The contraction of these myosin cables propels the oriented intercalation of the mesoderm cells (Rozbicki et al., 2015; Saadaoui et al., 2020; Serra et al., 2021 preprint). Once the mesoderm progenitors are located in the epiblast midline, they undergo an EMT and ingress as individual cells into the embryo generating the primitive streak (Nakaya and Sheng, 2008; Voiculescu et al., 2014) (Fig. 2Cb). All tissue movements are abolished when the mesoderm induction is blocked with a pan FGF receptor inhibitor (Chuai et al., 2023), which shows that the mesoderm cells are the motor of the tissue flows during avian gastrulation.

### Ring-shaped mesoderm with cell ingressions

Adding FGF at the hypoblast side induces the expansion of the mesoderm territory along the periphery of the epiblast (Alev et al., 2013; Chuai et al., 2023). In this configuration, myosin cables extend along the long axis of the mesoderm, i.e. tangentially to the mesoderm ring (Chuai et al., 2023; Serra et al., 2021 preprint). Cells located in this ectopic mesoderm ring degrade the basement membrane, ingress and intercalate similarly to an elongating primitive streak (Chuai et al., 2023; Serra et al., 2021 preprint). After ingression, these cells acquire a migratory phenotype and migrate towards the ‘animal pole’, located in the central epiblast (Chuai et al., 2023) (Fig. 2Cc). Interestingly, both the cell ingression of cells along the circular periphery of the embryo and the consequent migration towards the centre of the epiblast (Chuai et al., 2023) are reminiscent of zebrafish gastrulation (Fig. 3A), where cells ingress along the germ ring and migrate towards the animal pole (Kimmel et al., 1990; Pinheiro et al., 2022; Pinheiro and Heisenberg, 2020).

### Ring-shaped mesoderm without cell ingressions

BMP inhibitors provide another avenue to expand the mesoderm territory from the crescent to a ring, presumably because the BMP signals no longer prevent the differentiation of the mesoderm in the anterior epiblast (Arias et al., 2017; Bertocchini and Stern, 2012; Chuai et al., 2023; Streit et al., 1998). Interestingly, the mesoderm cells created in this way cannot undergo proper EMT and individual ingression. Instead, they stay connected in a single-layered epithelial sheet. The mesoderm cells express myosin cables oriented tangentially to the ring (Serra et al., 2021 preprint). In these conditions, the mesoderm cells intercalate along the ring producing the contraction of the mesoderm territory. Once the mesoderm ring cannot be further compacted, the central epiblast buckles at the mesoderm-epiblast interface – probably due to the outward stress (Box 1) imposed by the epiboly of the area opaca. The buckling tissue folds inside the embryo, and the continuing intercalation of the mesoderm cells contract the ring fold further, resulting in the invagination of the central epiblast and part of the mesoderm ring (Chuai et al., 2023; Serra et al., 2021 preprint) (Fig. 2Cd). This process strongly resembles the involution and closing of the blastopore seen in *Xenopus* (Shook and Keller, 2008b); however, chick embryos invaginate the central epiblast, which generally would give rise to the neural plate (Fig. 2Cd), whereas *Xenopus* engulfs the vegetal portion of the embryo containing the yolk-enriched endoderm (Fig. 3B). This difference in behaviour is probably due to the mechanical impediments imposed by the large yolk and the area opaca (that attaches to the vitelline membrane, but see ‘A mesoderm-ring to -crescent transition is necessary for the evolution of large yolks’). Interestingly a similar phenotype has been observed in mutant mouse embryos with defective BMP signalling. Deletion of the BMP receptors ActRIIA and ActRIIB (Song et al., 1999) or the ActRIA (ALK2) receptor (Gu et al., 1999) results in the formation of smaller embryos, likely a result of a reduction of cell proliferation. It also results in a disruption of mesoderm formation, thickening of the ectoderm and a notable tissue ‘constriction’ at the interface of the embryonic and extra-embryonic domains. These embryo morphologies strongly resemble the phenotype of chick embryos in the presence of BMP inhibitors (Chuai et al., 2023), which suggests that large tissue contractions of the epiblast tissues following BMP inhibition might be a conserved behaviour in amniote embryos.

### Crescent-shaped mesoderm without cell ingressions

Finally, it is also possible to block cell ingression through the primitive streak via the inhibition of VEGF signalling (Chuai et al., 2023). Interestingly, blocking apical contraction and cell ingression in the primitive streak does not interfere notably with the directed intercalation of mesoderm cells. Instead, they accumulate in the midline of the embryo and, when the epiblast cannot accommodate any more tissue as the cells do not ingress, the streak epiblast buckles creating an invagination at the anterior tip (Fig. 2Ce). Similarly, two recent reports have shown that tissue buckling is possible in the absence of apical contraction, thanks to external forces in the surrounding tissues during fly gastrulation (Fierling et al., 2022; Guo et al., 2022) (Box 1). The invagination of the anterior tip of the streak resembles chameleon gastrulation, where a limited amount of the mesoderm is internalised through cell ingression at the posterior blastoporal plate and the rest through involution in the blastoporal canal in a more anterior position (Stower et al., 2015). This suggests that reducing the cell ingression at the primitive streak generates a mechanical landscape that produces a structure that might resemble the blastoporal canal seen in the streak of reptiles (Chuai et al., 2023; Serra et al., 2021 preprint) (Fig. 3D). Interestingly, some reports in mouse embryos suggest that reducing the amount of cell ingression in the primitive streak through *FGF8^−/−^* (Sun et al., 1999) or *FGFR1^−/−^* (Ciruna et al., 1997; Yamaguchi et al., 1994) knockouts also results in the buckling of the epiblast. However, it is unclear whether this phenotype results from defects in apical contraction and cell ingression or because the mesoderm cells that manage to ingress are unable to migrate out of the streak and accumulate below (Ciruna and Rossant, 2001; Sun et al., 1999).

Although a comprehensive molecular characterisation of EMT and differentiation markers remains to be conducted in these different scenarios, available data suggest that treatments that result in the involution of the tissue correlate with the absence of apical contraction, cell ingression and degradation of the basement membrane (Chuai et al., 2023). This suggests that when EMT is restricted, but when flows in the epiblast continue, this leads to the buckling of the tissue (Box 1). Altogether these observations strongly suggest that varying two different factors – the size and shape of the mesoderm (e.g. crescent to a ring) and the ability of the cells to undergo cell ingression – can reproduce crucial aspects of the gastrulation morphologies typical of vertebrate embryos.

## Evolution and constraints on amniote gastrulation

The experiments described above suggest that the shape of the mesoderm domain relative to the embryo topology and the internalisation mode are two major determinants of gastrulation morphology in very different animals. However, despite these insights and experimental validations of the molecular and cellular mechanisms that shape gastrulation, the evolutionary pressures and innovations that propelled the appearance of distinct gastrulation modes still need to be solved.

### A mesoderm-ring to -crescent transition is necessary for the evolution of large yolks

Among the many evolutionary forces that have been invoked in the shaping of gastrulation, probably the best understood evolutionary force is due to the changes in the amount of yolk in the egg that has occurred many times during evolution (Arendt and Nübler-Jung, 1999; Shook and Keller, 2008a; Takeuchi et al., 2009). The consequences of increasing the yolk size have been extensively studied in the context of the evolution of the amniote egg. Amniotes evolved from ancestors that could lay eggs on land, achieved through the acquisition of a mineralised shell that provided mechanical protection and three extra-embryonic membranes (Starck et al., 2021). Most importantly, the yolk experienced a significant increase in size, probably to increase the egg's energy depot, allowing the embryo to hatch as a miniature version of the adult instead of as a larva (Packard and Seymour, 1997).

**Fig. 4.**
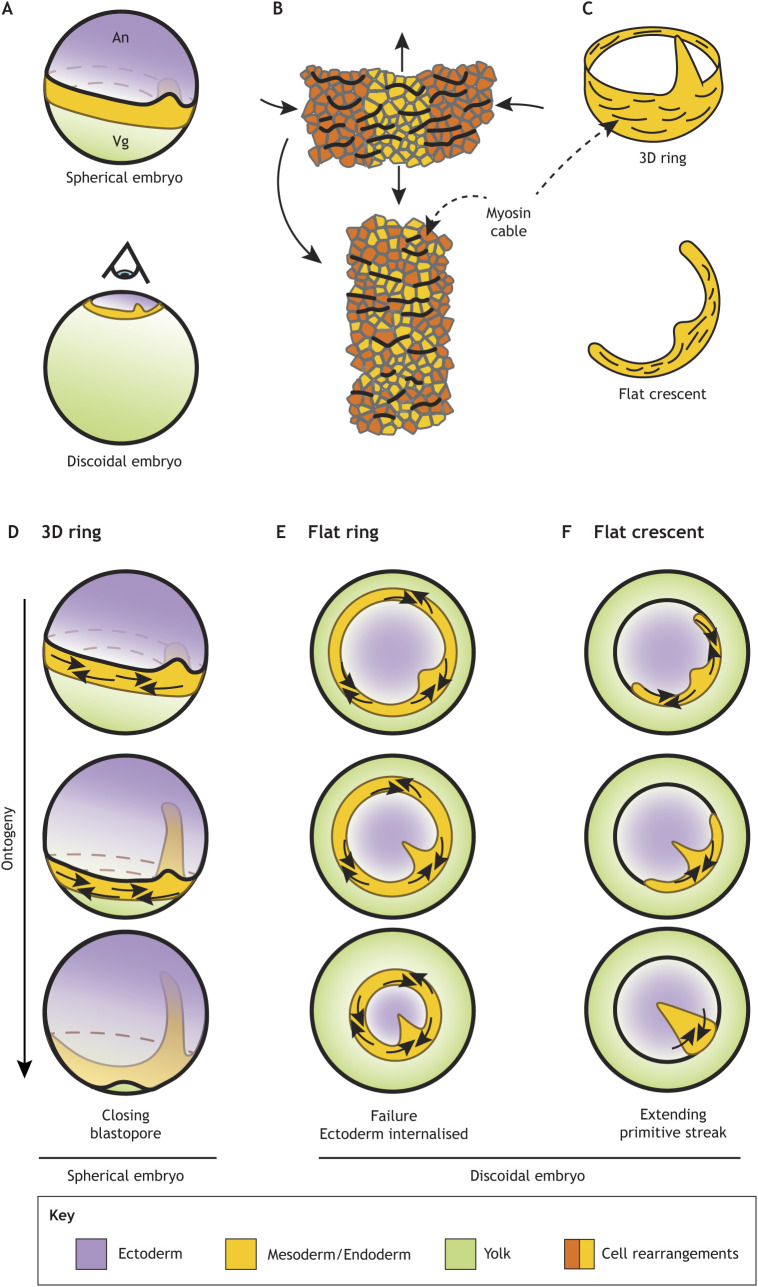
**Mesoderm-ring to -crescent transition.** (A) The increase in yolk size resulted in the flattening of the embryos during amniote evolution. The eye indicates the direction of the projection in E,F. (B) The direction of actomyosin cables is perpendicular to the direction of intercalation. Directed cell intercalation drives the convergent extension of tissue. (C) Actomyosin cables are organised along the long axis of the prospective mesoderm in tetrapods. (D-F) Mesoderm undergoes convergent extension in three different scenarios. In a 3D ring, the contraction of the ring does not interfere with the ectoderm expansion (D). A flat mesoderm ring undergoing convergent extension traps the ectoderm, which prevents its expansion (E). A flat mesoderm crescent undergoing convergent extension naturally collapses into the midline (F).

The increase in the amount of yolk generated mechanical and topological constraints that required adaptations in the gastrulation mode. The evolution of a large increase in yolk interfered with early development. The early cleavages became exclusively meroblastic due to the impossibility of splitting the enormous yolk cell (Cooper and Virta, 2007; Starck et al., 2021), leading to the formation of discoidal embryos that sit on top of the yolk (Fig. 4A). Furthermore, the large yolk impeded the complete internalisation of the yolk by epiboly, which blocked the closure of the blastopore (Arendt and Nübler-Jung, 1999; Starck et al., 2021).

In a seminal article on the evolution of the amniote gastrulation, Arendt and Nübler-Jung (1999) proposed that the meeting of the dorsal and ventral blastopore lips is a developmental requirement in discoidal amniote embryos that was achieved by a transition from a ring- to a crescent-shaped mesoderm domain. In this model, the ventral tips of the mesoderm crescent move towards the dorsal side of the blastopore creating two ‘mesodermal wings’ while the dorsal mesoderm involutes. It was suggested that the fusion of these mesodermal wings would form the blastoporal plate in ancient reptiles, such as turtles, while the involuting central portion constitutes the blastoporal canal (Fig. 3D). However, this model did not explain the tissue movements that drive the formation of the mesodermal wings. Furthermore, it was predicted that the meeting of mesodermal wings would leave a central suture line, but studies in turtles did not find such a structure in the blastoporal plate (Bertocchini et al., 2013; Coolen et al., 2008). The model was later revised and expanded (Shook and Keller, 2008a); instead of the expansion of the mesodermal wings, it was recognised that the collapse of the mesoderm crescent into the blastoporal plate was probably driven by the convergent extension of the tissue, which would not result in the formation of a midline suture. Here, we propose an alternative evolutionary constraint that drove the selection of crescent-shaped mesoderm over a ring-shaped domain based on the embryo topology and conserved tissue behaviours of the presumptive mesoderm.

### Flat ring constraint hypothesis for streak formation

The convergent extension of the mesoderm territory is most probably a conserved feature of chordate gastrulation that is necessary to establish the head-to-tail body axis in the developing embryo. This behaviour appears to be associated with the expression of brachyury*,* a mesoderm marker expressed around the blastopore in a highly conserved manner (Bruce and Winklbauer, 2020). Indeed, blocking brachyury expression often blocks blastopore closure, a process that involves the circumferential constriction and the simultaneous anteroposterior elongation of the tissue, generally associated with cell intercalation (Bruce and Winklbauer, 2020; Winklbauer, 2020). Furthermore, brachyury-positive mesoderm is associated with the formation of supracellular myosin IIb structures, likely because myosin IIb contraction is necessary to close the blastopore. In tetrapods, the contraction of the myosin IIb within the mesoderm appears to be organised in supracellular cables spanning several cells and extending along the long axis of the initial mesoderm domain, promoting oriented cell intercalation (Fig. 4B,C) as seen in *Xenopus* (Shindo and Wallingford, 2014) and chick embryos (Rozbicki et al., 2015). In teleost fish, an actomyosin mesh expressed in the yolk syncytial layer contracts the mesoderm ring and pulls the enveloping layer aiding in epiboly (Behrndt et al., 2012; Köppen et al., 2006). Therefore, brachyury expression appears to be generally associated with supracellular myosin structures that are crucial for closing the blastopore. However, in the very derived case of mouse gastrulation, brachyury expression appears to be dissociated from convergence extension in the primitive streak, and the mesoderm cells are already specified at the midline (Williams et al., 2012).

In spherical embryos, the closing of the mesoderm ring does not present a topological impediment for the formation of the ectoderm. In this scenario, the contracting mesoderm ring moves vegetally to form the posterior tissues, leaving the neural plate to form anteriorly (Fig. 4D). However, the flattening of the embryo, associated with the significant increases in yolk size, presented a topological problem for the development of the ectoderm, that is now trapped inside the flat ring of mesoderm in the same plane (Fig. 4E). The contraction of such a ring would impede the formation of head and dorsal tissues. The generation of mesoderm rings in the discoidal chick embryo supports this hypothesis (Chuai et al., 2023). As stated above, it is possible to induce mesoderm rings where the cells can undergo ingression or not (FGF activation or BMP inhibition, respectively). However, independently of the protocol used to induce mesoderm rings, the ectopic mesoderm presents myosin cables extending along the long axis of the mesoderm (tangentially to the ring) (Chuai et al., 2023). In the presence of cell ingression (FGF activation), the ectoderm remains on the surface, but the mesoderm ring prevents its expansion (Fig. 2Cc), whereas in the absence of cell ingression (BMP inhibition) the ring contracts, producing the invagination of the prospective ectoderm and neural plate (Fig. 2Cd). Preventing the growth of the head tissues or internalising them will fail to produce viable embryos. Therefore, before the evolution of large yolks that flattened the blastoderm, amniote embryos had to transition from a mesoderm ring to a mesoderm crescent, where the neural plate is induced anteriorly, effectively separated from the mesoderm (Fig. 4F). Indeed, the computation of the ‘attractors’ and ‘repellers’ that guide the tissue flows during chick gastrulation reveals the presence of a repeller that bisects the posterior two-thirds of the marginal zone, leaving space for the ectoderm to grow anteriorly (Serra et al., 2020; Serrano Nájera and Weijer, 2020). Furthermore, if brachyury expression, and thus the capacity to undergo convergent extension, is conserved in this flat mesoderm crescent, all the mesoderm territory will automatically converge into a flat triangular structure on the midline, similar to the blastoporal plate of reptiles.

We propose that a crescent-shaped mesoderm is topologically necessary for expanding the anterior body structures when the embryonic tissues cannot complete epiboly and close the blastopore during gastrulation. Indeed, other animals that experienced a great increase in the yolk that impeded the process of involution also possess flat embryos characterised by a crescent-shaped mesoderm restricted to the posterior side of the embryo, as in cartilaginous fish, such as the catshark (Fig. 3C) (Coolen et al., 2007; Sauka-Spengler et al., 2003).

### The evolution of the mesoderm-ring to -crescent transition

We propose that the mesoderm ring-crescent transition is only necessary when the size of the yolk impedes blastopore closure. Our prediction is that spherical embryos that can engulf the yolk through epiboly and accomplish blastopore closure will present a ring of mesoderm characterised by brachyury expression at the rim of the blastopore. The completion of epiboly depends on the relative size of the yolk and the number of cell layers in the animal pole that can contribute to spread the blastoderm through radial cell intercalation (Keller, 1980). Indeed, when the size of the blastoderm in zebrafish embryos is reduced, cutting out a piece of the yolk improves the rate of the embryos that complete epiboly and close the blastopore (Ishimatsu et al., 2018). The evolution of extra-embryonic tissues can also help to support the engulfing of the yolk, such as the enveloping layer of zebrafish (Concha and Reig, 2022). Interestingly, in amniotes, the mesoderm ring-to-crescent transition coincides with the evolution of extra-embryonic tissues that support the engulfing of the yolk later in development (Arendt and Nübler-Jung, 1999). Furthermore, at least in some discoidal embryos, brachyury expression is transiently initiated as a ring and later is restricted to a posterior crescent, as in catshark (Coolen et al., 2007) and mouse (Perea-Gomez et al., 2004). Finally, amniotes only initiate gastrulation when the embryo has a large number of cells, which could constitute a conserved trait used to complete epiboly in amniote ancestors.

As predicted, the mesoderm-ring to -crescent transition is not necessary to evolve a large yolk, if this does not prevent the closing of the blastopore, as observed in rainbow trout (Finch et al., 2010), the marsupial frog (del Pino, 1996); and the coquí frog (del Pino et al., 2007; Ninomiya et al., 2001). These organisms maintain a ring of brachyury expression and exhibit a range of other changes, such as modified developmental timing and the dissociation of notochord formation and blastopore closure, that allows them to close the blastopore in the presence of a large yolk.

Probably, the evolution of flat embryos with crescent-shaped mesoderm could only occur from spherical ancestors with crescent-shaped mesoderm. In fact, a crescent-shaped mesoderm does not create topological problems for spherical embryos. For example, both lampreys (Takeuchi et al., 2009) and urodele amphibians (Kaneda et al., 2009) present holoblastic cleavage and a derived brachyury expression that concentrates in a dorsal crescent during most of the gastrulation period, and only closes into a ring during blastopore closure (Bruce and Winklbauer, 2020). In these embryos, involution only occurs through the dorsal blastopore and ingression in the lateral mesoderm (Shook et al., 2002; Shook and Keller, 2008a). Therefore, urodele amphibians already display a gastrulation mode that might facilitate the mesoderm-ring to -crescent transition. This way, in a urodele-like ancestor, the increase in the yolk would impede blastopore closure, producing loss of the final annular pattern of brachyury expression, but would not constrain the evolution of a flat gastrulation mode because the mesoderm is already organised as a crescent.

Thus, increases in yolk size have likely been the most significant evolutionary force in early development, which imposed difficulties to complete epiboly and, in extreme cases, constrained a three-dimensional spherical embryo into a two-dimensional discoidal embryo, with extra-embryonic tissues on the outside. This way, early embryonic movements must adapt to the different mechanical and topological environments imposed by the yolk to permit the development of dorsal structures. However, those constraints disappear once the embryo finishes gastrulation and is big enough to be separated from the yolk. These more permissive conditions do not require further modification of the embryonic shape and possibly explain the conservation of the pharyngula stage among very distinct phyla (Steventon et al., 2021).

### The evolution of the primitive streak from a crescent-shaped mesoderm

Birds and mammals evolved primitive streaks independently of each other in a case of convergent evolution from the blastoporal plate and canal of their reptilian ancestors (Stower and Bertocchini, 2017). Reptiles present a bimodal gastrulation, where the mesoderm is internalised through involution in the blastoporal canal and ingression in the blastoporal plate (Bertocchini et al., 2013). Therefore, the evolution of the primitive streak required entirely substituting involution as a tissue internalisation mechanism with individual cell ingression (Arendt and Nübler-Jung, 1999; Bertocchini et al., 2013; Sheng et al., 2021; Shook and Keller, 2008a; Stower and Bertocchini, 2017).

The comparison of morphogenetic events and cell fate between embryos of different species suggests that embryonic mesoderm (axial, paraxial, intermediate and lateral plate) is internalised through the blastoporal canal. In contrast, the extra-embryonic mesoderm is internalised by cell ingression in the blastoporal plate (Bertocchini et al., 2013). This suggests that the reptilian blastoporal canal is homologous with the two-thirds most anterior (dorsal) section of the primitive streak, including the Hensen's node, a structure with organising properties homologous to the dorsal blastoporal lip (Bertocchini et al., 2013). In contrast, the blastoporal plate corresponds to the posterior third of the streak. Interestingly, moderate concentrations of VEGF signalling inhibitors only block cell ingression in the posterior region of the primitive streak, whereas higher concentrations act along on its length (Chuai et al., 2023). Furthermore, the expression of VEGF receptors accumulates in the posterior third of the primitive streak (Chuai et al., 2023). The extra-embryonic mesoderm is an amniote invention, which might have evolved alongside cell ingression as its internalisation mode in the blastoporal plate. Early extra-embryonic mesoderm has an essential role in erythropoiesis, so it makes sense that its specification requires VEGF signalling (a factor necessary for vasculogenesis, angiogenesis and haematopoiesis) (Liang et al., 2001). It is tempting to hypothesise that VEGF was only required in the extra-embryonic mesoderm in early reptiles. Later, it was acquired as an ingression signal by embryonic mesoderm in birds during the transition towards a primitive streak gastrulation mode.

Finally, it has been suggested that the increase in the length of the midline (from the reptilian bimodal blastopore to the avian primitive streak) was required to increase the speed of internalisation (Shook and Keller, 2008a). However, the primitive streak is created by the combination of oriented cell intercalation and cell ingression. Although cell intercalation only contributes to the convergent extension of the tissue, cell ingressions might counteract the extension because this process removes cells from the epiblast that otherwise would participate in convergent extension and because apical contraction of individual cells in the chick is likely an isotropic process. However, blocking cell ingression does not result in a longer axis (Chuai et al., 2023), but instead generates a shorter, wider streak territory with a small involution at its tip, as typically seen in reptiles (Fig. 3D). The buckling at the tip of the extending streak is caused by a failure of the streak epiblast to accommodate all the tissue flowing in from the mesoderm wings at the base of the streak and might resemble the reptilian blastoporal canal (Chuai et al., 2023). This suggests that the lengthening of the blastoporal plate into the primitive streak might not be directly selected, but is just a consequence of the addition of cell ingression and the mechanical environment; the cause and effect are currently unclear. This phenomenon also suggests that the convergent extension of the tissue in the absence of ingression could be necessary to fold the blastoporal canal in reptiles. Different amounts of cell ingression in the blastoporal plate might contribute to explaining the morphological differences between different reptilian gastrulae: the wider, less elongated blastoporal plate/canal in ancient reptiles, such as turtles (Coolen et al., 2008) could be associated with less cell ingression, whereas the elongated blastoporal plate/canal could be associated with higher rates of cell ingression, as in the chameleon (Stower et al., 2015).

## Evolvability of gastrulation

Gastrulation is an early event in embryonic development and mutations affecting gastrulation can propagate to later developmental stages, which could create a crucial evolutionary constraint. Surprisingly, gastrulation is very diverse, which suggests that this is a highly evolvable process. A phenotype is robust when many mutations are necessary to alter the phenotype. Robust phenotypes have higher evolvability because they can accumulate a greater genotypic diversity (Wagner, 2008). The original gastrulation programme probably was a relatively robust phenotype because, as demonstrated in cnidarians (Kirillova et al., 2018), as long as the endoderm is internalised, development can proceed to the adult stage, independently of the internalisation mode (ingression or invagination). This multiplicity of possible cell behaviours leading to mesoderm internalisation provides enough flexibility to evolve new gastrulation morphologies. The same occurs with other cell behaviours. For example, mesoderm contraction and blastopore closure in zebrafish, *Xenopus* and chick occur through different mechanisms. However, the mesoderm is characterised by brachyury expression in all three organisms.

Although the cell behaviours during gastrulation can differ significantly among different organisms, cell type markers, signalling pathways and fate maps are broadly conserved (Arendt and Nübler-Jung, 1999; Solnica-Krezel, 2005). These suggest that the cell behaviours that generate the stresses and define the material properties of the tissues generated and, in turn, define the shape of the gastrula, are more evolvable than the GRNs that pattern them (Shook and Keller, 2008b). One possible reason is that, although morphogenetic changes are local in space and time, the changes in GRNs might propagate through developmental time, generating problems for later events. This suggests that it is possible to modify cell behaviours during evolution, minimally affecting the GRNs. This might be plausible, as variations in the embryo geometry or relatively small changes in maternal mRNA loading or signalling can strongly impact cell behaviours resulting in significant morphogenetic variations, as demonstrated by experimental manipulations that generate new gastrulation modes in the same organism (Fig. 2). This non-linearity between changes in signalling/patterning and the morphogenetic programme carried out by cell behaviours might have favoured gastrulation evolvability.

## Conclusion and outlook

The synthesis of alternative gastrulation modes now achieved in a variety of diverse organisms (Chuai et al., 2023; Kirillova et al., 2018; Urbansky et al., 2016) is informative for the following reasons. (1) It shows which cell behaviours are tightly linked to differentiation. For example, in the chick embryo, it is possible to change the size and geometry of the mesoderm precursor domain and its internalisation mode; however, in all cases mesoderm formation is associated with the formation of supercellular myosin cables and cell intercalation, resulting in contraction along the long axis of the domain. (2) It can reveal developmental constraints previously not clearly recognised as well as the intrinsic benefits of specific internalisation modes. This has been clearly demonstrated by the generation of different contracting rings of mesoderm in the chick embryo and by the observation that, during fly gastrulation, invagination is faster and more robust than individual ingression. (3) It shows which developmental trajectories are available during gastrulation. For example, *N. vectensis* can access new morphogenetic modes when the embryo topology changes (Fig. 2A), whereas in flies, modifications in maternal mRNA loading of molecules controlling apical contraction and EMT are likely sufficient to generate alternative internalisation modes (Fig. 2B).

The experimental generation of gastrulation modes in embryos of selected organisms has successfully highlighted some of the evolutionary constraints associated with them. Interesting alternative approaches include a bottom-up approach to study developmental constraints during gastrulation using *in vitro* embryo-like systems. These are usually created through the aggregation of multipotent epiblast cells (reviewed by Anlas and Trivedi, 2021; Emig and Williams, 2022; Schauer and Heisenberg, 2021). These systems demonstrate that differentiation of the germ layers can proceed in the absence of specific mechanical constraints. However, their morphogenetic potential when devoid of a physical substrate is limited (Sheng et al., 2021). The progressive addition of specific mechanical constraints and substrates to these systems might constitute another approach to understanding the limitations and the evolution of morphogenesis in the early embryo.

Alternatively, the study of other non-model organisms also might cast some light on other possible trajectories during the evolution of gastrulation. Gastrulation probably appeared when the patterning of an epithelial embryo became associated with the morphogenetic movements that internalise the endoderm and mesoderm layers; however, in some cases, gastrulation might have become dissociated from the epithelial cell movements. That could be the case with the very highly derived developmental programmes in tunicates, such as salps (Piette and Lemaire, 2015; Winkley et al., 2020), killifish (Pereiro et al., 2017) and planarians (Alvarado, 2003). Indeed, flatworms could be an especially relevant model for the study of the interactions between the yolk and the early embryo, as they present very different egg geometries, where in different species the yolk is below, inside and surrounding the blastomeres, but they exhibit a ‘phylotypic stage’ (Martín-Durán and Egger, 2012). Therefore, these organisms could indicate where and how the constraints imposed by the yolk are relaxed during development.

Altogether, the ability to generate alternative gastrulation modes in either selected organisms or in synthetic systems opens a new avenue to study the crucial processes and selective pressures that shape the evolution of gastrulation across the animal kingdom.
